# Supplementary material for: To strike or not to strike? an investigation of the determinants of strike participation at the Fridays for Future climate strikes in Switzerland
Source: PLoS One. 2021 Oct 14;16(10):e0257296. doi: 10.1371/journal.pone.0257296 (PMC8516297; doi:10.1371/journal.pone.0257296)
Supplement: S1 File — (DOCX) [file pone.0257296.s001.docx]

S1

**S1 Table. Scales used for analyses**

| Scale | Item |
| --- | --- |
| Climate change worry (α = .78)  1 = *do not worry at all*, 6 = *very worried* | I am worried that climate change will have a strong impact on…   1. Sea-level rise 2. Loss of biodiversity 3. Health-related risks 4. Precipitation patterns 5. Frequency and magnitude of extreme weather events 6. Climate migration |
| Trust in governments (α = .75)  1= *do not trust at all*, 6 = *trust completely* | 1. How much to you trust the world’s governments to cooperate and dedicate resources in order to act on climate change? 2. How much to you trust the Swiss government to act on climate change? |
| Protest enjoyment (α = .70)  1 = *strongly disagree*, 6 = *strongly agree* | 1. In general, I enjoy going on the streets to protest. 2. I enjoy being part of a movement. 3. I like challenging the established order. |

**Knowledge about climate change**

To determine the students’ objective levels of knowledge regarding climate change, we used a shortened version of the knowledge scale developed by Tobler et al. (2012) that was subsequently validated by Shi et al. (2016). The scale comprises three knowledge dimensions, namely knowledge about the physical characteristics of climate change, about the causes and about the consequences of climate change. Participants had to indicate whether the statements provided about climate change were correct, incorrect or whether they didn’t know. ‘Don’t know’ answers were subsequently coded as incorrect, resulting in dichotomous data (1 = *correct*, 0 = *incorrect*). Cronbach’s alphas were unacceptably low for the physical characteristics ($\alpha$ = 0.49), the causes of climate change ($\alpha$ = 0.29) and the consequences of climate change ($\alpha$ = 0.37). The scalability of the knowledge scale was further assessed with a Mokken scale analysis (MSA). An MSA is a probabilistic, non-parametric equivalent of the Guttman scaling process used to assess the scalability of scales with dichotomous answer options. We were only able to obtain a scale based on four items that had sufficiently high scalability coefficients (H > 3); however, the reliability remained low ($\alpha$ = 0.54). This low reliability can be explained by the fact that the scale was constructed for use with nationally representative samples, whereas we used it with a very specific sub-population, namely university students. Therefore, the level of climate change knowledge was higher in our sample than in a nationally representative Swiss sample from a study conducted in 2016 that used the same scale (Shi et al., 2016). For the nationally representative Swiss sample, the proportions of correct responses in relation to knowledge about the physical characteristics, the causes, and the consequences of climate change were 50%, 59% and 75%, respectively, while the values in our sample were 81%, 70% and 91%, respectively. Thus, the knowledge levels concerning climate change were very high in our sample, possibly due to ceiling effects. Given insufficient reliability, we were unable to use the knowledge scales for subsequent analyses.

**References**

Shi, J., Visschers, V. H. M., Siegrist, M., & Arvai, J. (2016). Knowledge as a driver of public perceptions about climate change reassessed. *Nature Climate Change*, *6*(8), 759–762. https://doi.org/10.1038/nclimate2997

Tobler, C., Visschers, V. H. M., & Siegrist, M. (2012). Consumers’ knowledge about climate change. *Climatic Change*, *114*(2), 189–209. https://doi.org/10.1007/s10584-011-0393-1

**English Questionnaire**

Questionnaire study
„Climate strike“

I participate in this study on a voluntary basis and can withdraw from the study at any time without giving reasons and without any negative consequences.

Yes  No

Please read the following points carefully:

- Please answer the questions spontaneously.
- Please answer the questions in the order they are written out. You may feel that some questions are similar. This is intentionally, and we would like to ask you to answer all questions.
- The obtained data will only be used for scientific research. Your responses will be treated strictly confidential. The statistical analyses will be conducted in such a way that individuals cannot be identified, and no conclusions can be drawn to individuals.
- Please only choose one answer option per question, unless mentioned otherwise.
- If you have marked the wrong box as an answer option, please follow the instructions below.

X

wrong answer right answer

### If you have any questions regarding this study, you can contact us by writing an e-mail to: gea.hoogendoorn@hest.ethz.ch or viktoria.cologna@hest.ethz.ch

**In the following part you will find several statements related to the topic of climate change. Please indicate whether you think these statements are true or false. If you are unsure, please tick «Don’t know».**

|  | **True** | **False** | **Don’t know** |
| --- | --- | --- | --- |
| Burning oil produces CO2. |  |  |  |
| CO2 is harmful to plants. |  |  |  |
| Nuclear power plants emit CO2 during operation |  |  |  |
| At the same quantity, CO2 is more harmful to the climate than methane. |  |  |  |
| The global CO2 concentration in the atmosphere has increased during the past 250 years |  |  |  |
| Climate change is mainly caused by human activities |  |  |  |
| The last century’s global increase in temperature was the largest during the past 1,000 years. |  |  |  |
| Today’s global CO2 concentration in the atmosphere has already occurred in the past 650’000 years. |  |  |  |

**For the next decades, the majority of climate scientists expect...**

|  | **True** | **False** | **Don’t know** |
| --- | --- | --- | --- |
| A warmer climate to increase the melting of polar ice, which will lead to an overall rise of the sea level. |  |  |  |
| An increase in extreme events, such as droughts, floods and storms. |  |  |  |
| A warmer climate to increase water evaporation, which will lead to an overall decrease of the sea level. |  |  |  |
| The climate to change evenly all over the world. |  |  |  |

|  | **Do not agree at all** |  |  |  |  | **Completely agree** |
| --- | --- | --- | --- | --- | --- | --- |
| I worry that the state of the climate is changing. |  |  |  |  |  |  |
| Climate change has severe consequences for humans and nature. |  |  |  |  |  |  |
| Climate protection is important for our future. |  |  |  |  |  |  |
| We must protect the climate’s equilibrium. |  |  |  |  |  |  |

**To what extent do you agree with the following statements:**

**I am worried that climate change will have a strong impact on…**

|  | **Not worried at all** |  |  |  |  | **Very worried** |
| --- | --- | --- | --- | --- | --- | --- |
| Sea-level rise |  |  |  |  |  |  |
| Loss of biodiversity |  |  |  |  |  |  |
| Health-related risks |  |  |  |  |  |  |
| Changing precipitation patterns |  |  |  |  |  |  |
| The frequency and magnitude of extreme weather events |  |  |  |  |  |  |
| Human migration |  |  |  |  |  |  |

**Please answer the following questions:**

|  | **Do not trust at all** |  |  |  |  | **Trust completely** |
| --- | --- | --- | --- | --- | --- | --- |
| How much do you trust the world’s governments to cooperate and dedicate resources in order to act on climate change? |  |  |  |  |  |  |
| How much do you trust the Swiss government to act on climate change? |  |  |  |  |  |  |
| How much do you trust climate scientists to provide correct information on climate change? |  |  |  |  |  |  |

**To what extent do you agree with the following statements?**

|  | **Do not agree at all** |  |  |  |  | **Agree completely** |
| --- | --- | --- | --- | --- | --- | --- |
| It is just too difficult for someone like me to do much about the climate change. |  |  |  |  |  |  |
| My actions will influence others to behave in ways that mitigate the effects of climate change. |  |  |  |  |  |  |
| My individual behavior can help or harm the environment. |  |  |  |  |  |  |
| Humans can’t reduce climate change. |  |  |  |  |  |  |
| Humans could reduce climate change, but people aren’t willing to change their behavior, so we’re not going to. |  |  |  |  |  |  |
| Humans could reduce climate change, but it’s unclear at this point whether we will do what’s needed. |  |  |  |  |  |  |
| Humans can reduce climate change, and we are going to do so successfully. |  |  |  |  |  |  |

**To what extent do you agree with the following statements?**

|  | **Do not agree at all** |  |  |  |  | **Agree completely** |
| --- | --- | --- | --- | --- | --- | --- |
| In general, I enjoy going on the streets to protest. |  |  |  |  |  |  |
| I enjoy being part of a movement. |  |  |  |  |  |  |
| I like challenging the established order. |  |  |  |  |  |  |

**Please answer the following question:**

|  | **Not successful at all** |  |  |  |  | **Very successful** |
| --- | --- | --- | --- | --- | --- | --- |
| How successful do you think the climate strikes will be in making a difference? |  |  |  |  |  |  |

**Please indicate how often you perform the following activities:**

|  | **Once** | **Twice** | **Three times** | **Four times** | **Five times** | **More than five times** | **Never** |
| --- | --- | --- | --- | --- | --- | --- | --- |
| How often do you eat meat per week? |  |  |  |  |  |  |  |
| How often did you fly in 2017 and 2018? |  |  |  |  |  |  |  |
|  | **None** | **1** | **2** | **3** | **4** | **More than 4** |  |
| How many of these flights were intercontinental flights? |  |  |  |  |  |  |  |

|  | **Yes** | **No** |  |
| --- | --- | --- | --- |
| If you did fly in the last two years, did you pay to compensate your emissions? |  |  |  |
| Did you donate money to an environmental organization last year? |  |  |  |
| Have you ever participated in a climate strike? |  |  |  |
| Did you participate in more than one climate strike? |  |  |  |
|  | **Yes** | **No** | **Don’t know** |
| Are you planning in the future to take part at the next climate strike? |  |  |  |

**What is your birthyear?**

..................................................................................................

**Please indicate your gender**

Female  Male  other

**At what level are you currently studying?**

Bachelor  Master  PhD

**What do you study?**

..................................................................................................

**Did you have university classes on Friday during the Spring’19 semester?**

**yes**  no

**If yes, were they compulsory?**

yes  no

**What is your political orientation?**

0 Strongly left wing – 100 strongly right-wing

## Thank you for participating in this study! Do you want to receive the results of this study? Please write down your e-mail address below!

**German Questionnaire**

Fragebogen zur Studie
„Klimastreik“

Ich nehme an dieser Studie freiwillig teil und kann jederzeit ohne Angabe von Gründen meine Zustimmung zur Teilnahme widerrufen, ohne dass für mich deswegen Nachteile entstehen.

Ja  Nein

Bitte beachten Sie folgende Punkte:

- Beantworten Sie die Fragen spontan.
- Beantworten Sie die Fragen der Reihe nach. Möglicherweise werden Sie den Eindruck haben, dass einige Fragen ähnlich sind. Dies ist absichtlich der Fall, bitte beantworten Sie trotzdem jede Frage.
- Die gewonnenen Daten werden ausschliesslich zur wissenschaftlichen Forschung und Lehre verwendet. Ihre Antworten werden streng vertraulich behandelt. Die statistischen Auswertungen lassen keine Rückschlüsse auf Einzelpersonen zu.
- Kreuzen Sie jeweils nur ein Antwortkästchen pro Frage an, ausser es wird explizit anders gesagt.
- Falls Sie versehentlich ein falsches Kästchen angekreuzt haben, so malen Sie dieses Kästchen aus und kreuzen sie das richtige Kästchen an.

X

Falsche Antwort Richtige Antwort

### Falls Sie Fragen zu dieser Studie haben, können Sie uns unter folgenden Email-Adressen erreichen: [gea.hoogendoorn@hest.ethz.ch](mailto:gea.hoogendoorn@hest.ethz.ch) oder [viktoria.cologna@hest.ethz.ch](mailto:viktoria.cologna@hest.ethz.ch)

**Bitte geben Sie an, ob diese Aussagen Ihrer Meinung nach richtig oder falsch sind.**

Falls Sie sich nicht sicher sind, kreuzen Sie bitte «ich weiss nicht» an.

|  | **Richtig** | **Falsch** | **Weiss nicht** |
| --- | --- | --- | --- |
| Bei der Verbrennung von Öl wird CO_2_ produziert. |  |  |  |
| CO_2_ ist für Pflanzen schädlich. |  |  |  |
| Kernkraftwerke stossen während des Betriebs CO_2_ aus. |  |  |  |
| Bei gleicher Menge ist CO_2_ für das Klima schädlicher als Methan |  |  |  |
| Der weltweite CO_2_-Gehalt in der Atmosphäre hat in den letzten 250 Jahren zugenommen. |  |  |  |
| Der Klimawandel wird hauptsächlich durch menschliche Aktivitäten verursacht. |  |  |  |
| Der weltweite Temperaturanstieg im letzten Jahrhundert war der grösste wahrend der letzten 1‘000 Jahren. |  |  |  |
| Der heutige weltweite CO_2_-Wert in der Atmosphäre ist in den letzten 650‘000 Jahren bereits aufgetreten. |  |  |  |

**Für die nächsten Jahrzehnte erwartet die Mehrheit der Klimaforscher...**

|  | **Richtig** | **Falsch** | **Weiss nicht** |
| --- | --- | --- | --- |
| Ein wärmeres Klima, welches dazu führen wird, dass mehr Eis an den Polenkappen schmilzt, wodurch der Meeresspiegel insgesamt steigen wird. |  |  |  |
| Eine Zunahme von Extremereignissen wie Dürren, Überflutungen, Hochwasser und Stürmen. |  |  |  |
| Ein wärmeres Klima mit erhöhter Wasserverdunstung, welche dazu führt, dass der Meeresspiegel insgesamt sinken wird. |  |  |  |
| Dass sich das Klima weltweit gleichmässig verändern wird. |  |  |  |

**Wie sehr stimmen Sie folgende Aussagen zu?**

|  | **Stimme überhaupt nicht zu** |  |  |  |  | **Stimme voll und ganz zu** |
| --- | --- | --- | --- | --- | --- | --- |
| Ich mache mir Sorgen, dass sich der Zustand des Klimas ändert. |  |  |  |  |  |  |
| Der Klimawandel hat schwerwiegende Folge für Mensch und Natur. |  |  |  |  |  |  |
| Der Klimaschutz ist wichtig für unsere Zukunft. |  |  |  |  |  |  |
| Wir müssen das Gleichgewicht des Klimas schützen. |  |  |  |  |  |  |

**Ich mache mir Sorgen, dass der Klimawandel einen Starken Einfluss haben wird auf…**

|  | **Gar nicht besorgt** |  |  |  |  | **Sehr besorgt** |
| --- | --- | --- | --- | --- | --- | --- |
| den Meeresspiegel |  |  |  |  |  |  |
| die Biodiversität |  |  |  |  |  |  |
| die menschliche Gesundheit |  |  |  |  |  |  |
| die globalen Niederschlagsmuster |  |  |  |  |  |  |
| die Häufigkeit und Stärke von Extremwetterverhältnissen |  |  |  |  |  |  |
| Migration von Menschen |  |  |  |  |  |  |

**Bitte beantworten Sie die folgenden Fragen:**

|  | **Gar kein Vertrauen** |  |  |  |  | **Absolutes Vertrauen** |
| --- | --- | --- | --- | --- | --- | --- |
| Wie stark vertrauen Sie internationalen Regierungen, dass diese kooperieren und Ressourcen aufwenden für den Klimaschutz? |  |  |  |  |  |  |
| Wie stark vertrauen Sie der Schweizer Regierung, sich für den Klimaschutz  einzusetzen? |  |  |  |  |  |  |
| Wie stark vertrauen Sie KlimawissenschaftlerInnen, korrekt über den Klimawandel zu berichten? |  |  |  |  |  |  |

**Wie sehr stimmen Sie folgenden Aussagen zu?**

|  | **Stimme überhaupt nicht zu** |  |  |  |  | **Stimme voll und ganz zu** |
| --- | --- | --- | --- | --- | --- | --- |
| Es ist zu schwierig für jemanden wie mich viel gegen den Klimawandel zu tun. |  |  |  |  |  |  |
| Meine Taten werden andere dazu beeinflussen sich so zu verhalten, dass die Auswirkungen des Klimawandels gemildert werden. |  |  |  |  |  |  |
| Mein eigenes Verhalten kann der Umwelt schaden oder ihr auch helfen. |  |  |  |  |  |  |
| Menschen können den Klimawandel nicht mildern. |  |  |  |  |  |  |
| Menschen könnten den Klimawandel mildern, aber niemand ist dazu bereit, sein Verhalten zu ändern, und darum wird sich auch nichts ändern. |  |  |  |  |  |  |
| Menschen könnten den Klimawandel mildern, aber es ist nicht sicher, ob wir so handeln werden wie benötigt. |  |  |  |  |  |  |
| Menschen können den Klimawandel reduzieren und das werden wir auch erfolgreich schaffen. |  |  |  |  |  |  |

**Wie sehr stimmen Sie folgenden Aussagen zu?**

|  | **Stimme überhaupt nicht zu** |  |  |  |  | **Stimme voll und ganz zu** |
| --- | --- | --- | --- | --- | --- | --- |
| Im Allgemeinen protestiere ich gerne. |  |  |  |  |  |  |
| Es gefällt mir Teil einer Bewegung zu sein. |  |  |  |  |  |  |
| Ich hinterfrage den Status Quo. |  |  |  |  |  |  |

**Wie sehr stimmen Sie folgender Aussage zu?**

|  | **Überhaupt nicht erfolgreich** |  |  |  |  | **Sehr erfolgreich** |
| --- | --- | --- | --- | --- | --- | --- |
| Wie erfolgreich werden die Klimastreiks darin sein, einen Unterschied zu machen? |  |  |  |  |  |  |

**Bitte geben Sie an, wie oft Sie folgende Aktivitäten durchführen:**

|  | **1 mal** | **2 mal** | **3 mal** | **4 mal** | **5 mal** | **Mehr als 5 mal** | **Nie** |
| --- | --- | --- | --- | --- | --- | --- | --- |
| Wie oft essen Sie in der Woche Fleisch? |  |  |  |  |  |  |  |
| Wie häufig sind Sie 2017 und 2018 geflogen? |  |  |  |  |  |  |  |
|  | **Keiner** | **1** | **2** | **3** | **4** | **Mehr als 4** | |
| Wie viele dieser Flüge waren interkontinentale Flüge? |  |  |  |  |  |  | |

|  | | **Ja** | **Nein** |
| --- | --- | --- | --- |
| Falls Sie in den letzten zwei Jahren geflogen sind, haben sie für CO_2_ Kompensationen gezahlt? | |  |  |
| Haben Sie im letzten Jahr Geld an eine Umweltorganisation gespendet? | |  |  |
| Haben Sie jemals an einem Klimastreik teilgenommen? | |  |  |
| Haben Sie an mehr als einem Klimastreik teilgenommen? | |  |  |
|  | **Ja** | **Nein** | **Weiss nicht** |
| Haben sie vor, am nächsten Klimastreik teilzunehmen? |  |  |  |

**Was ist Ihr Geburtsjahr?**

..................................................................................................

**Bitte geben Sie Ihr Geschlecht an.**

Weiblich  Männlich  anderes

**Ich studiere zurzeit für einen...**

Bachelorabschluss  Masterabschluss  PhD

**Was ist Ihr Studiengang / Studiengebiet?**

..................................................................................................

**Haben Sie in FS2019 Vorlesungen belegt, die Freitagnachmittag stattfanden?**

**Ja**  Nein

**Falls ja, war Anwesenheit an diesen Veranstaltungen verpflichtend?**

Ja  Nein

**Wie würden Sie Ihre politische Einstellung einstufen?**

Sehr links mitte Sehr rechts
